# CYP2E1 and Parkinson’s disease in a MPTP-induced C57BL/6 mouse model

**DOI:** 10.1186/1750-1326-8-S1-P9

**Published:** 2013-09-13

**Authors:** Rahman Basaran, Elcin Deniz Ozdamar, Benay Can-Eke

**Affiliations:** 1 Ankara University Faculty of Pharmacy Department of Toxicology, Ankara, Turkey

## 

In recent years, extensive investigations on neurodegenerative disorders has been providing considerable information about the relationship between environmetal toxins and CYP450 enzymes. A chemical neurotoxin, MPTP (1-methyl-4-phenyl-1,2,3,6 tetrahydropyridine), can lead to Parkinson’s disease in C57BI/6 mouse model. CYP2E1 is a substantial enzyme involved in the metabolism of xenobiotics in brain and liver. It’s also known to generate reactive oxygen species and promote oxidative stress causing many diseases such as Parkinson’s Disease, Alzheimer Disease, atherosclerosis. CYP2E1 is expressed in brain less than liver however brain CYP2E1 contributes to progression of diseases. Evidence for involvement of CYP2E1 in the MPTP induced mouse model of Parkinson’s Disease has been reported. In the present study, brain and liver CYP2E1 activities are compared between control and parkinsonism groups of C57BI/6 and Swiss albino mice. CYP2E1 protein expressions are evaluated by the method of Western blotting in these goups. Also brain and liver CYP2E1 activities and protein expressions are compared between Swiss albino and C57BI/6 mice. In this study, we monitored various profiles for different mouse species in the context of CYP2E1 activities and expressions.

